# From Symptom to Outcome: Defining Clinically Meaningful Patient‐Reported Appetite Loss in Non‐Small‐Cell Lung Cancer

**DOI:** 10.1002/jcsm.70150

**Published:** 2025-12-03

**Authors:** Jiawei Zhou, Benyam Muluneh, Quefeng Li, Lynne I. Wagner, Yuchen Wang, Jim H. Hughes

**Affiliations:** ^1^ Division of Pharmacotherapy and Experimental Therapeutics, Eshelman School of Pharmacy University of North Carolina at Chapel Hill Chapel Hill North Carolina USA; ^2^ Lineberger Comprehensive Cancer Center, School of Medicine University of North Carolina at Chapel Hill Chapel Hill North Carolina USA; ^3^ Gillings School of Global Public Health University of North Carolina at Chapel Hill Chapel Hill North Carolina USA; ^4^ Pfizer Inc New York New York USA

**Keywords:** appetite, clinical meaningful thresholds, non‐small‐cell lung cancer, patient‐reported outcomes

## Abstract

**Background:**

Appetite loss is a common and distressing symptom in non‐small‐cell lung cancer (NSCLC), driven by both treatment side effects and disease progression. It often leads to unintended weight loss and cancer cachexia, significantly impairing patients' quality of life and survival. Yet, appetite loss remains under‐recognized in oncology care, with no standard assessment tools or universal management guidelines. In this study, we developed a predictive model to identify clinically meaningful thresholds of appetite loss that were associated with significant weight reduction and decreased survival. We aim to highlight the clinical consequences of appetite loss and advocate for more patient‐centred treatment strategies that address this often overlooked but impactful symptom in cancer care.

**Methods:**

We analysed longitudinal patient‐reported appetite scores, measured by the Lung Cancer Symptom Scale (LCSS), and body weight data from 476 NSCLC patients receiving supportive care and recovering from prior chemoradiotherapy (recovering cohort). A mechanism‐based population modelling approach was used to predict the impact of appetite changes on body weight, accounting for significant data variability. Model validation was conducted using data from 380 NSCLC patients undergoing docetaxel chemotherapy (chemotherapy cohort). Clinically meaningful appetite loss thresholds were determined based on the model‐predicted appetite loss associated with a 3.5‐kg body weight loss and significantly worse survival (hazard ratio > 1, *p* < 0.05).

**Results:**

We found that, according to the LCSS (100‐mm visual analogue scale), a 30‐mm (90% CI, 28–32) improvement in appetite corresponded to a 3.5‐kg weight gain in the recovering cohort, while a 23‐mm (90% CI, 17–30) decline correlated with a 3.5‐kg weight loss in the chemotherapy cohort. Significant associations were observed between appetite loss trajectories and overall survival (*p* < 0.001). Clinically meaningful thresholds for appetite loss were identified as 4 mm at 1 month and 11 mm at 3 months, both significantly associated with reduced OS in patients receiving docetaxel chemotherapy (*p* < 0.05).

**Conclusions:**

We developed a population predictive model to characterize the relationship between patient‐reported appetite and body weight, identifying clinically meaningful thresholds for appetite loss. This work highlights the importance of managing appetite loss in oncology care and supports its use as a quantitative endpoint in clinical trials and practice.

## 1 Introduction

Lung cancer remains the leading cause of cancer‐related mortality in the United States, accounting for an estimated 124 730 deaths in 2025 [1]. Non‐small‐cell lung cancer (NSCLC) accounts for more than 80% of all lung cancer cases and the 5‐year survival rate is only 26.4% [2]. In addition to poor prognosis, NSCLC patients often experience a high symptom burden—including cough, pain, fatigue and appetite loss—driven by both the disease and treatment‐related toxicity. These symptoms significantly impair their quality of life and daily functioning [3].

Among these symptoms, appetite loss is particularly prevalent and distressing, affecting approximately 60%–70% of lung cancer patients [4]. Persistent appetite loss can lead to unintentional weight loss, malnutrition and cancer cachexia—a syndrome characterized by severe muscle wasting, metabolic abnormalities and increased mortality [5, 6]. Despite its high prevalence and clinical consequences, appetite loss remains under‐recognized and under‐managed in routine oncology practice. There are currently no standardized assessment tools or universally accepted evidence‐based guidelines for managing appetite loss [5]. Patients often report that this symptom significantly impacts their well‐being and quality of life, yet it receives relatively little attention in clinical care [7, 8].

This gap underscores an urgent need to better understand how appetite loss relates to downstream clinical outcomes, including weight reduction and overall survival (OS), and to develop targeted strategies to mitigate its impact. In this study, we address this unmet need by evaluating patient‐reported appetite loss during treatment in NSCLC and quantifying its relationship with body weight change and survival outcomes.

In our study, we focus on patient‐reported appetite loss rather than clinician‐reported adverse events, as it more accurately reflects patient experience and aligns with the growing emphasis on patient‐centred care in oncology [9, 10, 11]. However, patient‐reported outcomes (PRO) data present unique analytical challenges, including high variability and noise [12]. To address these challenges, we proposed a novel population modelling approach. Population modelling is a computational method used to find common patterns in data from many people, while also accounting for between‐subject variability and measurement noise. It is widely used in pharmaceutical research to understand how medicines behave in different patients [12]. In our prior work, we demonstrated that this approach could handle the variability and noise in the PRO data and predict survival based on patient‐reported symptoms [13].

In the current study, we extended this approach to model the mechanistic relationship between patient‐reported appetite and body weight using data from NSCLC patients. We further explored how changes in appetite are associated with patient OS. We aim to identify clinically meaningful thresholds of appetite loss associated with significant weight loss and worse survival outcomes. These thresholds may serve as early indicators of cancer cachexia risk. By linking subjective symptoms with objective outcomes, our study provides a quantitative framework to guide appetite monitoring and symptom management in NSCLC treatment. Ultimately, our goal is to highlight the clinical consequences of appetite loss and advocate for more patient‐centred treatment strategies that address this often overlooked but impactful symptom in cancer care.

## 2 Methods

### 2.1 Ethics Statement

This retrospective analysis used fully de‐identified data. The study was granted an Institutional Review Board (IRB) exemption by the University of North Carolina at Chapel Hill.

### 2.2 Data Source and Study Description

#### 2.2.1 Recovering Study Cohort

Appetite–body weight model was developed using data from a Phase III randomized, double‐blind clinical trial (NCT00409188) [14]. This trial enrolled patients with unresectable Stage III NSCLC who had completed chemoradiotherapy 4–12 weeks prior to randomization and had achieved either stable disease or an objective response. Participants included in our analysis were from the control arm, receiving supportive care such as psychosocial support and nutritional support, and were in the recovery phase following chemoradiation.

#### 2.2.2 Chemotherapy Study Cohort

To validate the model and workflow, we used data from another Phase III clinical trial (NCT00532155) [15]. This trial assessed the efficacy of aflibercept combined with docetaxel versus docetaxel alone in patients with advanced or metastatic nonsquamous NSCLC. Participants included in our analysis were from the control arm and received docetaxel at 75 mg/m^2^, administered intravenously over 1 h every 3 weeks, along with best supportive care.

Both trials were conducted in accordance with the International Conference on Harmonisation Good Clinical Practice (ICH‐GCP) guidelines and the Declaration of Helsinki. Study protocols were approved by local regulatory authorities and institutional ethics committees, and all participants provided written informed consent prior to enrollment.

### 2.3 Data Exclusions and Imputations

All control arm participants from both cohorts were included in the analysis. For each participant, patient‐reported Lung Cancer Symptom Scale (LCSS) scores [16], longitudinal body weight data, baseline demographics, clinical characteristics and OS outcomes were extracted. The patient‐reported LCSS consists of nine items measured using visual analogue scales (0–100 mm) to assess lung cancer symptom burden and quality of life [17, 18]. In our analysis, we focused on the appetite‐related item, ‘How is your appetite?’, where a score of 0 mm indicates ‘As good as it could be’ and 100 mm indicates ‘As bad as it could be’. Higher scores represent more severe appetite loss.

In the recovering study cohort, LCSS questionnaires were collected at baseline, Weeks 2, 5 and 8, then every 6 weeks from Week 13 until disease progression, with follow‐up assessments at 6 and 12 weeks post‐progression and every 12 weeks thereafter. Body weight was measured at baseline, weekly during Weeks 1–8, and every 6 weeks from Week 13 until progression [14]. In the chemotherapy study cohort, LCSS questionnaires were collected at baseline, Cycle 2, Cycle 4 and at the end of treatment. Body weight was measured at every treatment cycle [15].

Participants with only one appetite or body weight measurement were excluded from data analysis. No imputations were performed for missing data.

### 2.4 Appetite–Body Weight Model Development

To characterize the relationship between patient‐reported appetite and body weight, we developed a mechanism‐based model using longitudinal data from the recovering study cohort. The model captures underlying pharmacological mechanisms and disease progression in appetite loss and body weight reduction and consists of two components: the trajectory of appetite scores and their impact on body weight.

In the appetite model, patients in the recovery cohort showed early improvement in appetite as they recovered from chemoradiotherapy‐related side effects. As this early improvement could not be separated from any placebo effects [19], both recovery and placebo were described using an asymptotic exponential with *PMAX* and *Kp* parameters. The appetite loss due to disease progression was modelled using a separate exponential function, governed by a rate parameter, *SLP*. A higher score indicates greater appetite loss; therefore, appetite improvement components were modelled as reductions from the baseline, while appetite loss components were added to the baseline. The mathematical model for the appetite trajectory is presented in Equation (1).

(1) $$L\left(t\right)=L0+\left(e^{\mathrm{SLP}\cdot t}-1\right)-\text{PMAX}\cdot \left(1-e^{-\mathrm{Kp}\cdot t}\right)$$

where *L*(*t*) is the appetite scores change over time, *L*0 is the appetite score at baseline, *SLP* is the appetite loss rate, *PMAX* is the maximal appetite improvements and *Kp* is the appetite improvements offsite rate (1/day).

Changes in appetite were linked to changes in body weight, with the assumptions that appetite loss inhibits body weight gain through decreased food intake. An indirect response model was utilized to account for the physiological lag between changes in appetite and the corresponding changes in body weight [20]. The mathematical model for the appetite–body weight relationship is presented in Equation (2).

(2) $$\frac{d\;\mathrm{WT}}{\mathrm{dt}}=\mathrm{Kin}\cdot \left(1-\frac{\text{Imax}\cdot L\left(t\right)}{\mathrm{IC}50+L\left(t\right)}\right)-\text{Kout}\cdot \mathrm{WT}$$

where *WT* is body weight over time, *L*(*t*) is the appetite scores change over time (same as Equation (1)), *Kin* is the constant rate of body weight increase, *Kout* is the first‐order rate constant of body weight reduction, *Imax* is the maximum appetite loss impact on body weight reduction and *IC50* is the appetite loss that causes 50% *Imax*.

Additionally, the *Kout* parameter was set as Equation (3) to ensure a constant body weight if appetite does not change over time.

(3) $$\text{Kout}=\mathrm{Kin}\cdot \left(1-\frac{\text{Imax}\cdot L\left(0\right)}{\mathrm{IC}50+L\left(0\right)}\right)/\mathrm{WT}0$$

where *WT0* is the body weight at baseline and *L*(*0*) is the appetite score at baseline.

### 2.5 Population Modelling Approach

Given the substantial variability in the patient‐reported appetite scores, we used a population modelling approach to estimate the model parameters. This approach allows us to estimate parameter values that represent the average behavior of the overall population, in addition to values for each individual that capture how each participant's response differs from the average within the study cohort [12, 21]. We also tested whether covariates (demographics and baseline clinical characteristics) could help explain differences in appetite and body weight patterns between subjects [22]. To check model performance, we used goodness‐of‐fit plots and visual predictive checks (VPCs) [23, 24]. More details about this population modelling approach are provided in Supporting Information S1: Section 1.

### 2.6 Model Validation

We further validated the appetite–body weight model using data from the chemotherapy cohort. The model structure and parameters describing the relationship between appetite and body weight (*Kin*, *Imax* and *IC50* in Equation (2)) remained the same as the recovery cohort. However, parameters for the appetite trajectory (Equation (1)) and baseline body weight (*WT0*) were re‐estimated. These decisions assumed that while the effects of appetite on body weight are expected to be consistent across studies, the trajectory of appetite and body weight may not be the same, due to different study designs and participant populations.

### 2.7 Model Simulation

To evaluate the appetite–body weight relationship in the NSCLC population, the final model was used to simulate 1000 virtual studies, varying the model estimates according to parameter precision. Using the simulated appetite–body weight trajectories, we estimated the degree of appetite loss required to achieve a 3.5‐kg weight reduction—equivalent to 5% of body weight for a 70‐kg individual, a commonly used threshold in cancer cachexia trials [25], based on the chemotherapy cohort model parameters. Similarly, we estimated the amount of appetite improvements associated with a 3.5‐kg weight gain using the recovering cohort model parameters. Separate simulations were performed using individual parameters for each subject in the dataset to capture patient‐specific appetite and body weight trajectories. These results were used to assess the associations between appetite loss and survival outcomes.

### 2.8 Associations Between Appetite Trajectory and Survival

To assess the relationship between appetite trajectory and OS, we developed Cox proportional hazards models that incorporated both prognostic clinical characteristics and key appetite–body weight model parameters (*L0*, *PMAX*, *SLP*, *WT0* and *Imax*). The relative contribution of each parameter to survival was visualized using forest plots. Prognostic clinical variables were identified using the Least Absolute Shrinkage and Selection Operator (LASSO) algorithm, with fivefold cross‐validation. Variables were selected based on the *λ* value within one standard error of the minimum to ensure model robustness.

### 2.9 Clinically Meaningful Appetite Loss

Clinically meaningful appetite loss was defined as the level of appetite loss significantly associated with OS. Using model simulations from the chemotherapy study cohort, we obtained appetite scores change from baseline for each individual patient at 1 and 3 months after chemotherapy. Patients were then grouped by their appetite loss scores using cutoffs ranging from 0 to 15 mm. Cox proportional hazards models were constructed to compare the two groups, adjusting for confounding clinical variables selected by LASSO. The lowest cutoff yielding *p* value < 0.05 and hazard ratio (HR) indicating increased risk (HR > 1) was identified as the clinically meaningful threshold. A parallel analysis was conducted using body weight loss cutoffs to determine the level of weight reduction significantly associated with OS.

### 2.10 Statistical Analysis

Appetite trajectory parameter comparisons between the recovering and chemotherapy cohorts were performed using Wilcoxon tests. Differences in OS between groups were evaluated using Kaplan–Meier curves and compared with log‐rank tests. Associations between appetite trajectory parameters or appetite cutoffs with OS were evaluated using Cox proportional hazards models, with HR, 95% confidence intervals (CIs), and *p* values reported. A two‐sided *p* value < 0.05 was considered statistically significant.

### 2.11 Software and Code Availability

Population appetite–body weight model was developed using Monolix 2024R1. The model simulations were performed using Simulx 2024R1. Both Monolix and Simulx could be downloaded at https://lixoft.com/products/. Plots and survival analyses were performed using R 4.4.1 and RStudio Version 2022.07.1+554. The figures were reorganized in GraphPad Prism (Version 10.4.1). The model codes are provided in Supporting Information S1: Supplementary Codes.

### 2.12 Data Availability

The data were accessed through Project Data Sphere under licence in accordance with the Project Data Sphere Data Use Agreement [26]. The raw analysis data are available from the corresponding author upon reasonable request.

## 3 Results

### 3.1 Study Data and Workflow

**FIGURE 1:**
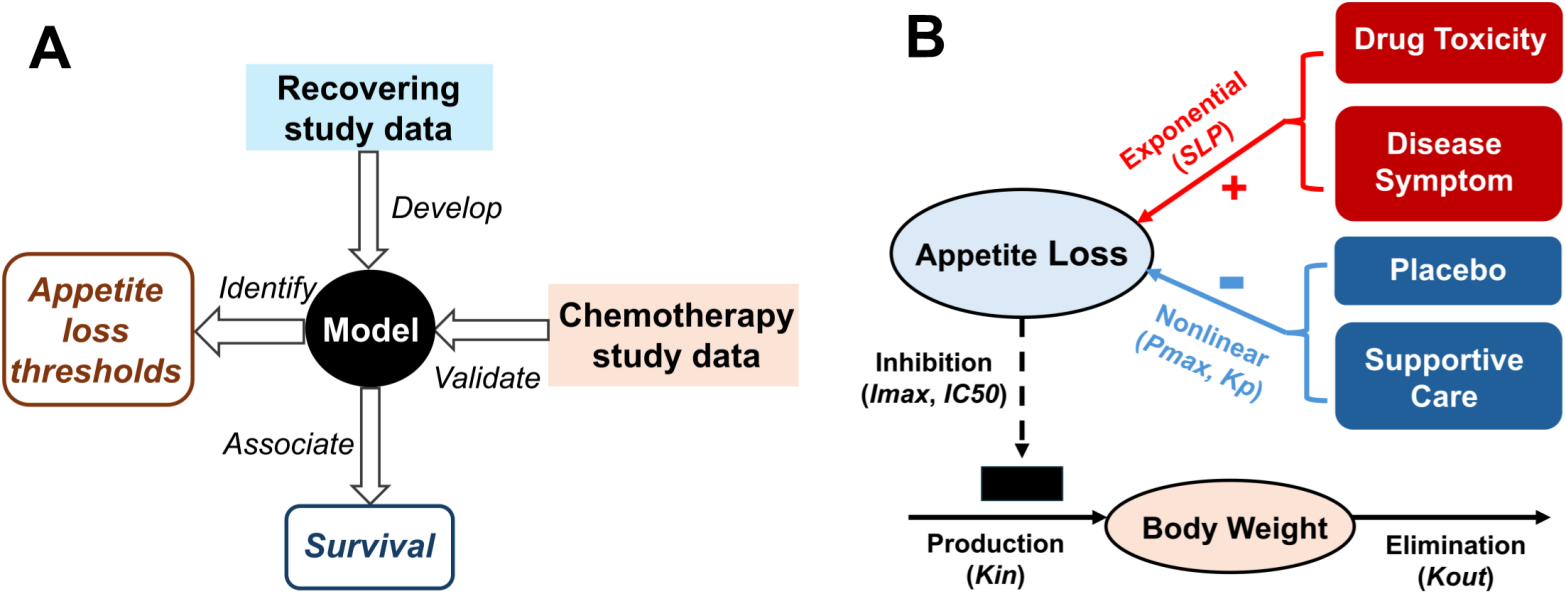
Study workflow and appetite–body weight model diagram. (A) The appetite–body weight model was developed using recovering study data and validated using chemotherapy study data. The model was used to identify clinically meaningful appetite loss thresholds and the associations between appetite loss and survival. (B) Appetite loss during treatment is influenced by both drug‐related toxicity and disease‐related symptoms, while placebo effects and supportive care can help improve appetite. In turn, reduced appetite limits body weight gain.

Data from two study cohorts (recovering and chemotherapy) were incorporated in our analysis and the study workflow is illustrated in Figure 1A. The recovering cohort included 476 participants, with 6517 patient‐reported appetite scores and 4284 body weight measurements. The chemotherapy cohort included 380 participants, contributing 1179 appetite scores and 3698 body weight observations (Figure S1). Participant demographics, baseline clinical characteristics and survival data are summarized in Table 1. Longitudinal trajectories of individual appetite scores and body weight changes from baseline are shown in Figure S2A–D. In the recovering cohort, a substantial proportion of participants experienced weight gain (Figure S2B), whereas most patients in the chemotherapy cohort showed weight loss over time (Figure S2D).

**TABLE 1:** Demographics of two study populations.

Trial	Recovering (*N* = 476)	Chemotherapy (*N* = 380)
Intervention	Placebo and supportive care	Docetaxel
Age, years	61 (24, 83)	61 (27, 80)
Sex		
Male	324 (68.1%)	243 (63.9%)
Female	152 (31.9%)	137 (36.1%)
Race		
White/Caucasian	441 (92.6%)	333 (87.6%)
Other	35 (7.4%)	47 (12.4%)
Height, cm	171 (144, 194)	168 (140, 193)
Missing	67 (14.1%)	0 (0%)
Weight, kg	74 (38.6, 140.8)	71 (35, 118.7)
Missing	69 (14.5%)	3 (0.8%)
Anxiety/depression history		
Yes	39 (8.2%)	39 (10.3%)
No	437 (91.8%)	341 (89.7%)
Smoking status		
Yes	443 (93.1%)	80 (21.1%)
No	33 (6.9%)	94 (24.7%)
Former smoker	Not Applicable	206 (54.2%)
ECOG performance status		
0	201 (42.2%)	130 (34.2%)
1	275 (57.8%)	232 (61.1%)
2	0 (0%)	18 (4.7%)
Overall survival, months	21 (1.7, 65.7)	11.7 (1.6, 36.1)
Missing	92 (19.3%)	9 (2.4%)
Disease stage		
I	0 (0%)	24 (6.3%)
II	0 (0%)	10 (2.6%)
III	476 (100%)	111 (29.2%)
IV	0 (0%)	223 (58.7%)
Missing	0 (0%)	12 (3.2%)
Response to initial chemoradiotherapy		Not applicable
Objective response	332 (69.7%)	
Stable disease	144 (30.3%)	
Type of initial chemoradiotherapy		Not applicable
Concurrent	289 (60.7%)	
Sequential	183 (38%)	
Missing	6 (1.3%)	

*Note:* Data are number (%) or median (range), unless otherwise specified.

Abbreviation: ECOG, Eastern Cooperative Oncology Group.

### 3.2 Characterizing Appetite–Body Weight Relationship in NSCLC

**FIGURE 2:**
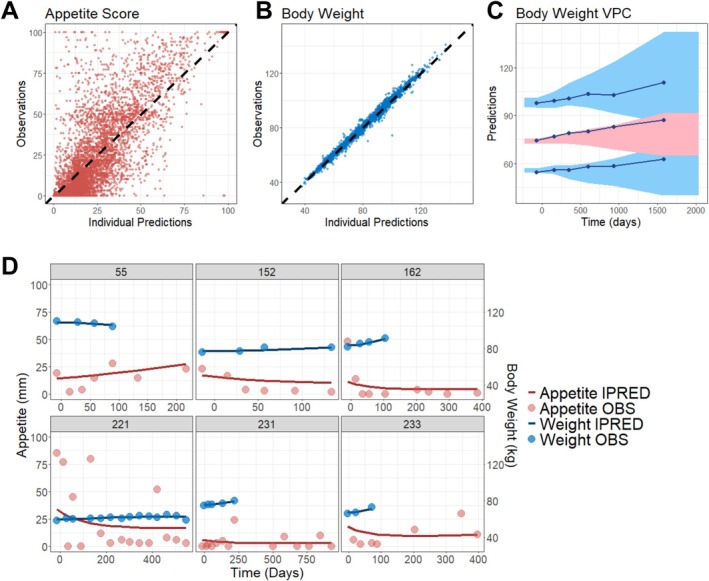
Final model captured appetite and body weight trajectories in recovering study. (A,B) Observations versus individual predictions of appetite scores (A) or body weight (B). The black dashed lines represent the lines of identify. (C) VPC of body weight over time. The observed data are represented by blue solid lines (median and 10th/90th percentiles). The simulated data based on the index population (1000 simulations) are represented by the red shaded area (90% PI of median) or blue shaded area (90% PI of 10th/90th percentiles). VPC has been corrected for dropout. (D) Plots of appetite scores and body weight over time for six randomly selected participants. Circles represent observed data, while solid lines represent individual model predictions. VPC, visual predictive check; PI, prediction interval; IPRED, individual predictions; OBS, observations.

The appetite–body weight model structure is illustrated in Figure 1B, where appetite loss due to drug toxicity and disease symptoms was modelled using an exponential function, while a separate asymptotic exponential component captured supportive care and placebo‐related improvements. Appetite loss, in turn, inhibited body weight gain via an indirect response model. A population modelling approach was used to estimate typical model parameters for the overall population, between‐subject variability, covariate effects and residual error based on data from the recovering cohort. Final model parameters are listed in Table S1. The appetite–body weight model adequately described the data, as shown by the alignment between observed and predicted values for appetite scores and body weight in Figure 2A,B. VPC of body weight change over time showed that observation data were well captured by model simulations (Figure 2C). Figure 2D presents model predictions overlaid with observations in six randomly selected subjects, demonstrating good alignment. These diagnostics confirm that the model appropriately captured the data from the recovering cohort.

We further validated the model using data from the chemotherapy study cohort using the same model structure and relationship between appetite and body weight (Table S1). The model successfully captured appetite and body weight data in the validation cohort (Figure S3A–D), indicating model generalizability across studies. In both the recovering and chemotherapy cohorts, ECOG performance status is a covariate on baseline appetite scores, with higher ECOG scores associated with poorer baseline appetite. Sex was identified as a covariate on baseline body weight, with female patients having 18.8% and 11.3% lower baseline weights than male patients in the recovering and chemotherapy cohorts, respectively (Table S1).

We further compared appetite trajectories between the two cohorts. Patients in the recovering cohort had significantly better baseline appetite (*p* < 0.0001, Figure S4A) and slower appetite decline (*p* < 0.0001, Figure S4B) compared to those in the chemotherapy cohort, which is consistent with expectations that patients tend to lose appetite due to chemotherapy side effects. While the absolute parameter values were beta‐transformed and not directly interpretable, the relative differences were consistent with clinical assumptions [27]. Interestingly, the maximum appetite improvement (*PMAX*) was higher in the chemotherapy cohort than in the recovering cohort and showed greater between‐subject variability, reflecting the heterogeneous responses and treatment‐related toxicities experienced by NSCLC patients during chemotherapy (Figure S4C).

**FIGURE 3:**
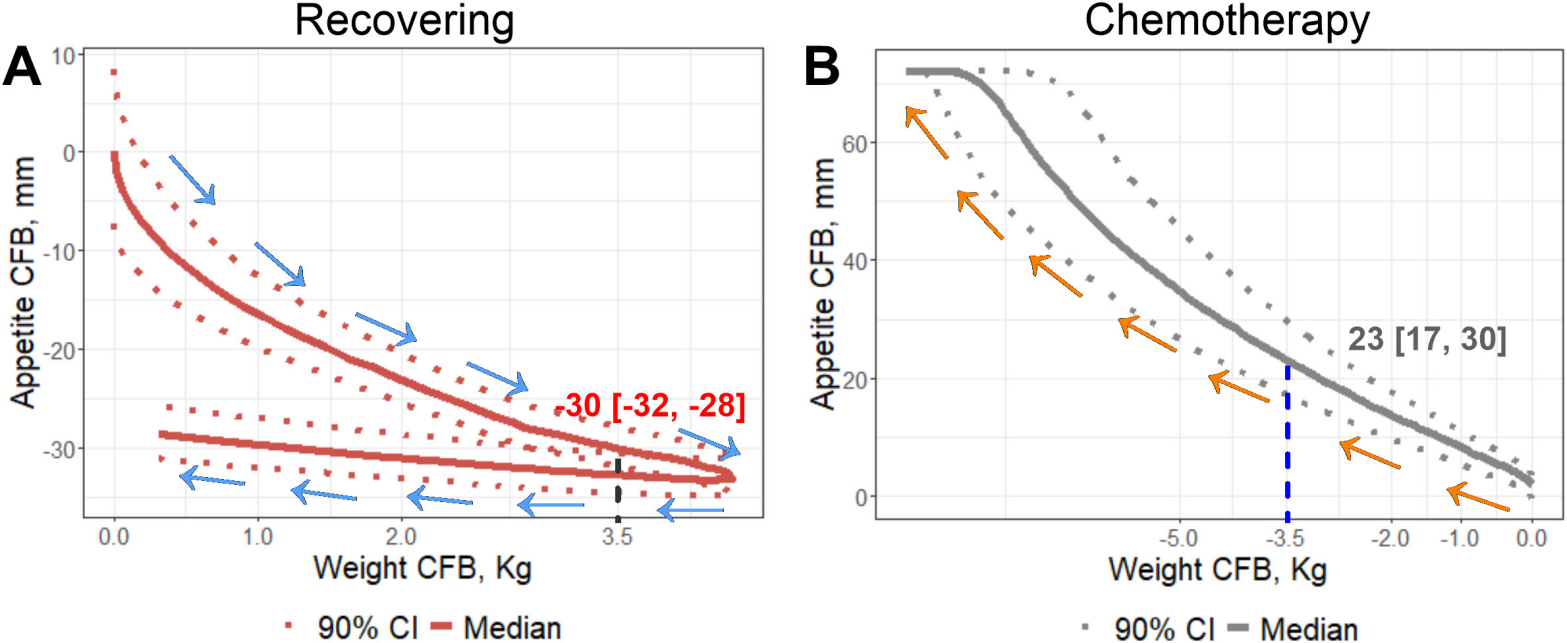
Quantitative relationship between appetite and body weight. (A) In the recovering study, appetite improvement leads to body weight gain, exhibiting a hysteresis loop due to a time delay. A median decrease in appetite score of 30 (90% CI: 28, 32) corresponds to a 3.5‐kg weight increase. (B) In the chemotherapy study, appetite loss leads to body weight reduction. A median increase in appetite score of 23 (90% CI: 17, 30) corresponds to a 3.5‐kg weight loss. CFB, change from baseline; CI, confidence interval; mm, millimetre; kg, kilogramme.

Characterization of the appetite–body weight relationship was achieved by simulating NSCLC population appetite and body weight profiles (Figure 3). L.I.W receives institutional research funding from the National Cancer Institute and previously received personal fees from Celgene/Bristol Myers Squibb as a member of the Scientific Steering Committee for the Connect Multiple Myeloma patient registry. During recovery from chemoradiotherapy, NSCLC patients initially experienced appetite improvement, with a 30 mm (90% CI, 28–32) decrease in appetite score (on a 100‐mm scale, indicating improved appetite) corresponding to a 3.5‐kg weight gain. Around Day 600, the patients' appetite began to decline, followed by a reduction in body weight, possibly due to disease progression. The time delay between changes in appetite and subsequent changes in body weight forms a hysteresis loop (Figure 3A). In contrast, patients under docetaxel chemotherapy experienced persistent appetite loss. A 23 mm (90% CI, 17–30) increase in appetite score was associated with a 3.5‐kg body weight reduction (Figure 3B). These findings quantify the appetite–weight relationship in NSCLC and provide a quantitative framework to map appetite loss to corresponding weight loss in future cancer cachexia studies.

### 3.3 Linking Appetite Loss Trajectories to OS

**FIGURE 4:**
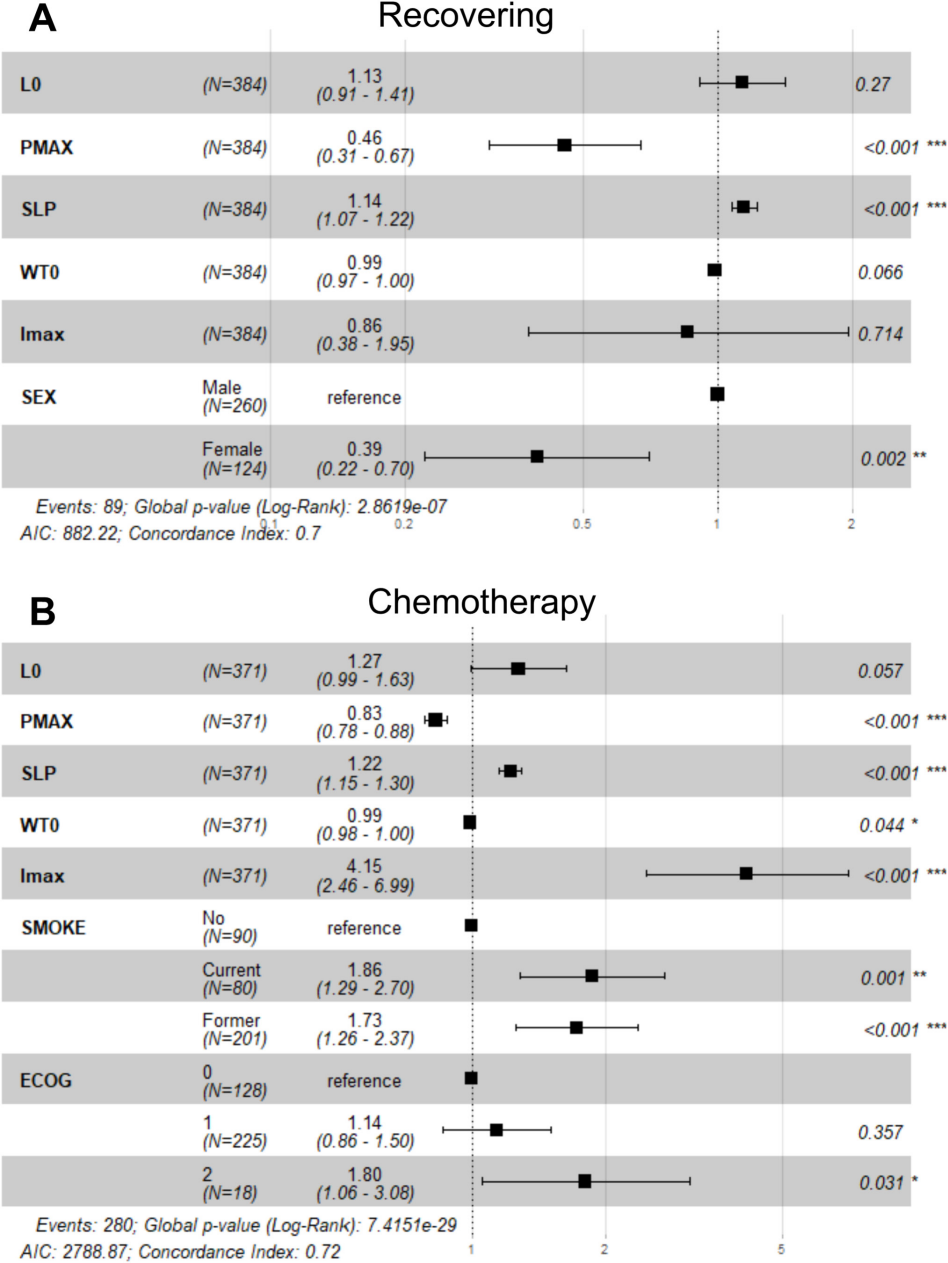
Associations between appetite–body weight model parameters and overall survival. Cox proportional hazards model incorporating model parameters and LASSO‐selected survival‐related clinical characteristics for recovering study cohort (A) or chemotherapy study cohort (B). The black boxes with horizontal error bars represent hazard ratio estimates with 95% CI. *p* values for each covariate are labelled on the right. LASSO, least absolute shrinkage and selection operator; CI, confidence interval; ECOG, Eastern Cooperative Oncology Group. Significance: *, *p* < 0.05; **, *p* < 0.01; ***, *p* < 0.001.

To explore the relationship between appetite loss trajectories and OS, Cox proportional hazards models were developed for both recovering and chemotherapy cohorts, incorporating key parameters from the appetite–body weight model. Prognostic covariates were selected using the LASSO algorithm. In the recovery cohort, sex was identified as a significant predictor of OS (Figure S4A), while in the chemotherapy cohort, smoking status and Eastern Cooperative Oncology Group (ECOG) performance status were identified (Figure S4B). After adjusting for these baseline prognostic factors, both *PMAX* (maximal appetite improvements) and *SLP* (appetite loss rate) remained significantly associated with OS. In the recovering cohort, a higher appetite loss rate (*SLP*) was associated with poorer survival (HR 1.14, 95% CI: 1.07–1.22), while greater improvements in appetite (*PMAX*) were linked to better survival (HR 0.46, 95% CI: 0.31–0.67) (Figure 4A). A similar pattern was observed in the chemotherapy cohort: Increased appetite loss was associated with poorer survival (HR 1.22, 95% CI 1.15–1.3) and stronger appetite improvements were associated with improved survival (HR 0.83, 95% CI 0.78–0.88) (Figure 4B).

### 3.4 Identifying Clinically Meaningful Appetite Loss Under Chemotherapy

**FIGURE 5:**
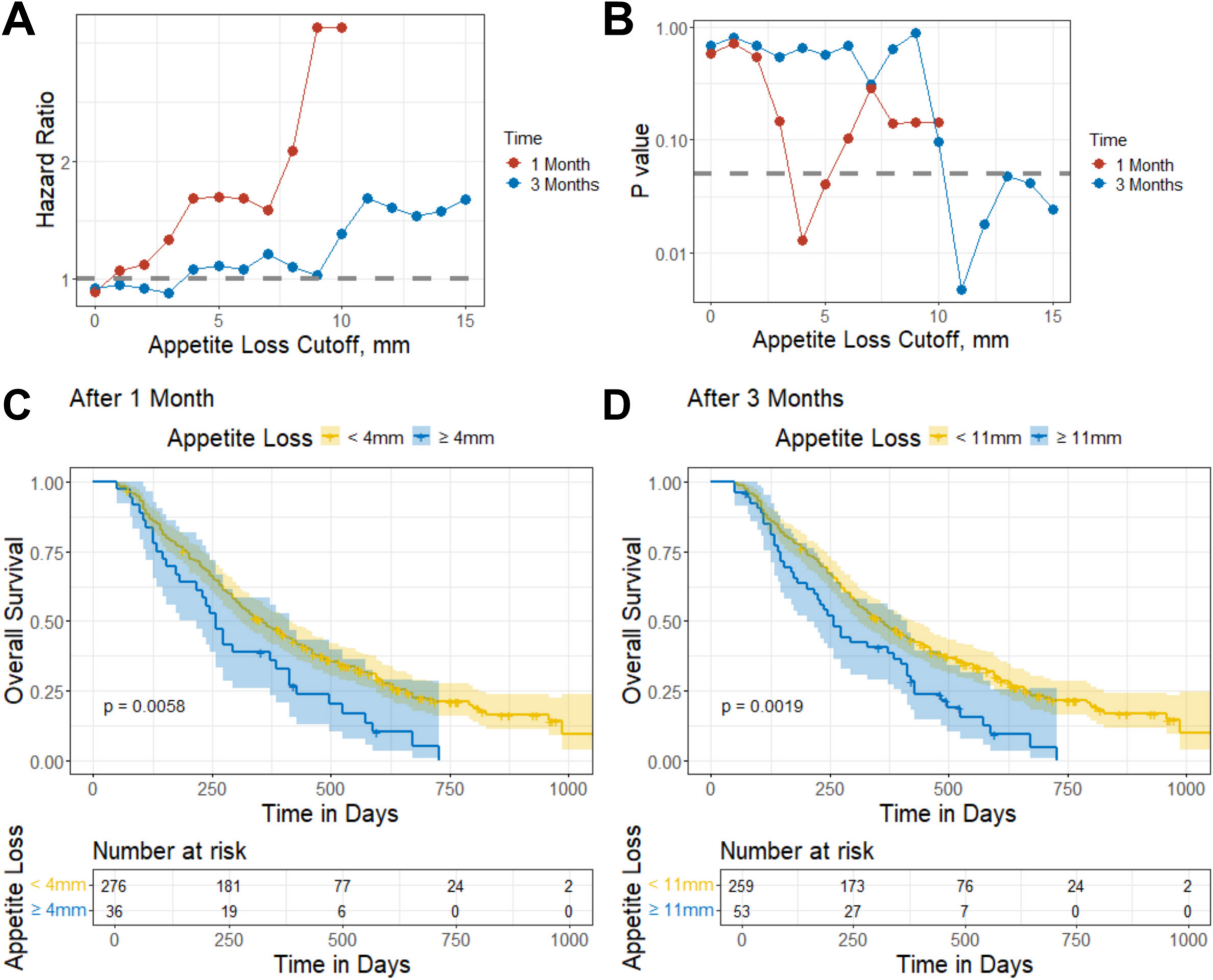
Clinically meaningful thresholds of appetite loss associated with overall survival in chemotherapy study cohort. (A,B) Hazard ratios (A) and *p* values (B) from Cox proportional hazards models assessing overall survival across varying appetite loss cutoffs after 1 or 3 months of chemotherapy. (C,D) Kaplan–Meier curves stratified by individual model‐predicted appetite loss: (C) using a 1‐month cutoff of ≥ 4 mm, associated with a hazard ratio (HR) of 1.68 (95% CI, 1.16–2.42), and (D) using a 3‐month cutoff of ≥ 11 mm, associated with an HR of 1.67 (95% CI, 1.20–2.31). A total of 237 events were observed. Shaded areas indicate 95% confidence intervals; *p* values are from log‐rank tests. mm, millimetre.

To identify clinically meaningful appetite loss associated with OS, patients were grouped based on their predicted appetite loss at 1 and 3 months after therapy, with cutoff values ranging from 0 to 15 mm tested. Each cutoff, along with prognostic variables identified via LASSO (smoking status and ECOG performance status), was included in Cox proportional hazards models. The resulting HRs and *p* values for each cutoff are shown in Figure 5A,B. The minimal cutoff associated with *p* value < 0.05 in the Cox proportional hazards model—4 mm at 1 month and 11 mm at 3 months—was selected as clinically meaningful thresholds. Kaplan–Meier curves based on 1 month and 3 months thresholds are shown in Figure 5C,D, respectively. Patients with appetite loss ≥ 4 mm at 1 month or ≥ 11 mm at 3 months had significantly worse OS (*p* < 0.05, HR > 1) compared to those with less appetite loss. Body weight reductions of 1 kg at 1 month and 3.5 kg at 3 months were identified as clinically meaningful body weight loss thresholds. Patients who exceeded these thresholds had significantly worse OS compared to those who did not (*p* < 0.05, Figure S5A–D).

## 4 Discussions

In this study, we developed a novel population modelling approach to quantify the relationship between patient‐reported appetite and body weight. On a 100‐mm scale, a 30‐mm improvement in appetite was associated with a 3.5‐kg weight gain in the recovering cohort, while a 23‐mm decline corresponded to a 3.5‐kg weight loss in the chemotherapy cohort. Appetite loss trajectories were also significantly associated with OS: Greater loss of appetite was related to worse survival, whereas greater improvement was linked to better outcomes. We identified clinically meaningful appetite loss thresholds—4 mm at 1 month and 11 mm at 3 months—that were significantly associated with OS in NSCLC patients receiving docetaxel chemotherapy. These thresholds may serve as early indicators of cancer cachexia risk and offer a quantitative basis for endpoint selection in future clinical trials.

In our study, we identified clinically meaningful appetite loss based on its significant association with worse OS (*p* < 0.05). The threshold we identified—an 11‐point decline after 3 months—is consistent with the commonly used 10‐point threshold in the LCSS Average Symptom Burden Index (ASBI) at 12 weeks in lung cancer clinical studies [28, 29]. Notably, our approach utilized a mechanism‐based model to define clinically meaningful appetite loss, offering a more sensitive and accurate alternative to traditional empirical endpoint selection. This provides a feasible and rational framework for selecting symptom‐based endpoints in future NSCLC clinical trials. In addition, our appetite–body weight model is generalizable across different NSCLC trial populations and can predict individual appetite loss trajectories and the corresponding body weight reduction based on early symptom data. This predictive framework offers the potential to identify patients at higher risk for developing cancer cachexia early in the treatment course.

The appetite–body weight model was initially developed using data from a cohort of patients recovering from chemoradiotherapy and was successfully validated in an independent cohort receiving docetaxel chemotherapy. Notably, patients in the recovery cohort showed better baseline appetite and slower appetite decline, underscoring the positive impact of supportive care in oncology [30, 31]. In contrast, the greater between‐subject variability in the maximal appetite improvement parameter observed in the chemotherapy cohort reflected the previously reported heterogeneity in treatment response and toxicity during chemotherapy [32, 33].

Our study has limitations. First, while appetite loss beyond the identified thresholds is expected to be associated with even smaller *p* values given a sufficient sample size, the current analysis shows a ‘bouncing back’ trend in *p* values (Figure 5B), likely due to the limited sample size in the chemotherapy cohort. To reduce the influence of sample size and improve generalizability, cutoff selection was guided not only by *p* values but also by the HR from the Cox proportional hazards model, which is less sensitive to sample size. Future analyses using larger datasets are needed to validate these thresholds. Second, our findings were derived using the LCSS instrument. For clinical trials that utilize other PRO tools, the differences in scales and response formats would require the development of instrument‐specific modelling approaches.

In summary, we developed a population model to characterize the relationship between patient‐reported appetite and body weight in NSCLC. We identified the appetite loss thresholds that were associated with 3.5‐kg body weight reduction and significantly worse survival (*p* < 0.05). These findings provide a quantitative framework to inform symptom‐based endpoints in future clinical trials and support early identification of patients at risk for cancer cachexia. By focusing on appetite loss, a frequently overlooked but clinically important symptom, our work supports a patient‐centred approach to improve symptom monitoring, guide supportive care strategies and ultimately enhance the quality of life for patients with NSCLC.

## Conflicts of Interest

B.M. may own stock in Novartis. Y.W. and J.H.H. are current employees of Pfizer and may own stock in Pfizer.

## Supporting information


**Table S1:** Final model parameter estimates.
**Figure S1:** Data inclusion and exclusion criteria for recovering dataset NCT00409188 (A) and chemotherapy dataset NCT00532155 (B).
**Figure S2:** Longitudinal appetite score and body weight change from baseline in Recovering study cohort (A‐B) and chemotherapy study cohort (C‐D). Each line represents one individual patient. CFB, change from baseline.
**Figure S3:** Final model captured appetite and body weight trajectories in chemotherapy study. (A‐B) Observations versus individual predictions of appetite scores (A) or body weight (B). The black dashed lines represent the lines of identify. (C) VPC of body weight over time. The observed data are represented by blue solid lines (median and 10th/90th percentiles). The simulated data based on the index population (1000 simulations) are represented by the red shaded area (90% PI of median) or blue shaded area (90% PI of 10th/90th percentiles). VPC has been corrected for dropout. (D) Plots of appetite scores and body weight over time for six randomly selected participants. Circles represent observed data, while solid lines represent individual model predictions. VPC, visual predictive check; PI, prediction interval; IPRED, individual predictions; OBS, observations.
**Figure S4:** Boxplots of appetite loss trajectory parameters comparing the recovering and chemotherapy cohorts. Individual patient‐level parameter estimates from the appetite–body weight model are shown for baseline appetite score L0 (A), appetite loss rate SLP (B), and maximal appetite improvement PMAX (C). Wilcoxon tests were used to assess differences between cohorts, with p‐values indicated above each plot. Boxes represent the interquartile range (IQR), with the horizontal line indicating the median. Whiskers extend to the smallest and largest values within 1.5 times IQR from the lower and upper quartiles; points outside this range are plotted as outliers.
**Figure S5:** The relationship between partial likelihood deviance and λ from LASSO algorithms for overall survival in recovering study cohort (A) and chemotherapy study cohort (B). The red dotted line represents the cross‐validation curve, and the grey error bars represent the upper and lower standard deviation curves along the lambda sequence. The selected lambdas (lambda min or lambda se) are indicated by the vertical dashed lines. λ = lamda.se were used to select covariates.
**Figure S6:** Clinically meaningful thresholds of body weight loss associated with overall survival in the chemotherapy study. (A–B) Hazard ratios (A) and p‐values (B) from Cox proportional hazards models assessing overall survival across varying body weight loss cutoffs after 1 or 3 months of chemotherapy. (C–D) Kaplan–Meier curves stratified by individual model‐predicted body weight loss: (C) using a 1‐month cutoff of ≥ 1 kg, associated with a hazard ratio (HR) of 2.78 [95% CI, 1.30–5.94], and (D) using a 3‐month cutoff of ≥ 3.5 kg, associated with an HR of 1.57 [95% CI, 1.00–2.46]. A total of 237 events were observed. Shaded areas indicate 95% confidence intervals; p‐values are from log‐rank tests; Kg, kilogram.

## References

[1] R. L. Siegel , T. B. Kratzer , A. N. Giaquinto , H. Sung , and A. Jemal , “Cancer Statistics, 2025,” Ca 75 (2025): 10–45. 39817679 10.3322/caac.21871 PMC11745215

[2] A. K. Ganti , A. B. Klein , I. Cotarla , B. Seal , and E. Chou , “Update of Incidence, Prevalence, Survival, and Initial Treatment in Patients With Non–Small Cell Lung Cancer in the US,” JAMA Oncology 7 (2021): 1824–1832. 34673888 10.1001/jamaoncol.2021.4932 PMC8532041

[3] J. Polanski , B. Jankowska‐Polanska , J. Rosinczuk , M. Chabowski , and A. Szymanska‐Chabowska , “Quality of Life of Patients With Lung Cancer,” Oncotargets and Therapy 9 (2016): 1023–1028. 27013895 10.2147/OTT.S100685 PMC4778772

[4] D. Kang , S. Kim , H. Kim , et al., “Surveillance of Symptom Burden Using the Patient‐Reported Outcome Version of the Common Terminology Criteria for Adverse Events in Patients With Various Types of Cancers During Chemoradiation Therapy: Real‐World Study,” JMIR Public Health and Surveillance 9 (2023): e44105. 36884274 10.2196/44105 PMC10034615

[5] R. A. Fielding , F. Landi , K. E. Smoyer , L. Tarasenko , and J. Groarke , “Association of Anorexia/Appetite Loss With Malnutrition and Mortality in Older Populations: A Systematic Literature Review,” Journal of Cachexia, Sarcopenia and Muscle 14 (2023): 706–729. 36807868 10.1002/jcsm.13186 PMC10067499

[6] S. Peixoto da Silva , J. M. Santos , M. P. Costa e Silva , R. M. Gil da Costa , and R. Medeiros , “Cancer Cachexia and Its Pathophysiology: Links With Sarcopenia, Anorexia and Asthenia,” Journal of Cachexia, Sarcopenia and Muscle 11 (2020): 619–635. 32142217 10.1002/jcsm.12528 PMC7296264

[7] D. E. B. Galindo , A. Vidal‐Casariego , A. Calleja‐Fernández , et al., “Appetite Disorders in Cancer Patients: Impact on Nutritional Status and Quality of Life,” Appetite 114 (2017): 23–27. 28315777 10.1016/j.appet.2017.03.020

[8] M. H. van den Beuken‐van , J. M. de Rijke , A. G. Kessels , H. C. Schouten , M. van Kleef , and J. Patijn , “Quality of Life and Non‐Pain Symptoms in Patients With Cancer,” Journal of Pain and Symptom Management 38 (2009): 216–233. 19564094 10.1016/j.jpainsymman.2008.08.014

[9] Optimizing the Dosage of Human Prescription Drugs and Biological Products for the Treatment of Oncologic Diseases, Draft Guidance for Industry, U.S. Food & Drug Administration. https://www.fda.gov/media/164555/download. accessed December 19, 2024.

[10] T. M. Atkinson , A. C. Dueck , D. V. Satele , et al., “Clinician Vs Patient Reporting of Baseline and Postbaseline Symptoms for Adverse Event Assessment in Cancer Clinical Trials,” JAMA Oncology 6 (2020): 437–439. 31876902 10.1001/jamaoncol.2019.5566 PMC6990818

[11] E. Basch , X. Jia , G. Heller , et al., “Adverse Symptom Event Reporting by Patients Vs Clinicians: Relationships With Clinical Outcomes,” JNCI Journal of the National Cancer Institute 101 (2009): 1624–1632. 19920223 10.1093/jnci/djp386 PMC2786917

[12] J. Zhou , B. Muluneh , Q. Li , and J. H. Hughes , “Revolutionizing Patient‐Reported Outcomes Analysis for Oncology Drug Development Using Population Models,” Clinical Cancer Research 31 (2025): 1580–1586. 40013954 10.1158/1078-0432.CCR-24-4073 PMC12010960

[13] J. Zhou , B. Muluneh , Z. Wang , H. Yao , and J. H. Hughes , “Leveraging Longitudinal Patient‐Reported Outcome Trajectories to Predict Survival in Non–Small Cell Lung Cancer,” Clinical Cancer Research 31 (2025): 2685–2694. 40272273 10.1158/1078-0432.CCR-25-0292

[14] C. Butts , M. A. Socinski , P. L. Mitchell , et al., “Tecemotide (L‐BLP25) Versus Placebo After Chemoradiotherapy for Stage III Non‐Small‐Cell Lung Cancer (START): A Randomised, Double‐Blind, Phase 3 Trial,” Lancet Oncology 15 (2014): 59–68. 24331154 10.1016/S1470-2045(13)70510-2

[15] R. Ramlau , V. Gorbunova , T. E. Ciuleanu , et al., “Aflibercept and Docetaxel Versus Docetaxel Alone After Platinum Failure in Patients With Advanced or Metastatic Non–Small‐Cell Lung Cancer: A Randomized, Controlled Phase III Trial,” Journal of Clinical Oncology 30 (2012): 3640–3647. 22965962 10.1200/JCO.2012.42.6932

[16] P. J. Hollen , R. J. Gralla , M. G. Kris , S. W. Eberly , and C. Cox , “Normative Data and Trends in Quality of Life From the Lung Cancer Symptom Scale (LCSS),” Supportive Care in Cancer 7 (1999): 140–148. 10335932 10.1007/s005200050244

[17] P. J. Hollen , R. J. Gralla , M. G. Kris , et al., “Measurement of Quality of Life in Patients With Lung Cancer in Multicenter Trials of New Therapies. Psychometric Assessment of the Lung Cancer Symptom Scale,” Cancer 73 (1994): 2087–2098. 8156514 10.1002/1097-0142(19940415)73:8<2087::aid-cncr2820730813>3.0.co;2-x

[18] P. J. Hollen , R. J. Gralla , M. G. Kris , and L. M. Potanovich , “Quality of Life Assessment in Individuals With Lung Cancer: Testing the Lung Cancer Symptom Scale (LCSS),” European Journal of Cancer 29 (1993): S51–S58. 10.1016/s0959-8049(05)80262-x 8381294

[19] G. Chvetzoff and I. F. Tannock , “Placebo Effects in Oncology,” Journal of the National Cancer Institute 95 (2003): 19–29. 12509397 10.1093/jnci/95.1.19

[20] W. J. Jusko and H. C. Ko , “Physiologic Indirect Response Models Characterize Diverse Types of Pharmacodynamic Effects,” Clinical Pharmacology and Therapeutics 56 (1994): 406–419. 7955802 10.1038/clpt.1994.155

[21] D. R. Mould and R. Upton , “Basic Concepts in Population Modeling, Simulation, and Model‐Based Drug Development,” CPT: Pharmacometrics & Systems Pharmacology 1 (2012): 1–14. 10.1038/psp.2012.4 PMC3606044 23835886

[22] M. M. Hutmacher and K. G. Kowalski , “Covariate Selection in Pharmacometric Analyses: A Review of Methods,” British Journal of Clinical Pharmacology 79 (2015): 132–147. 24962797 10.1111/bcp.12451 PMC4294083

[23] E. Comets , K. Brendel , and F. Mentré , “Model Evaluation in Nonlinear Mixed Effect Models, With Applications to Pharmacokinetics,” Journal de la Société Française de Statistique 151 (2010): 106–127.

[24] T. Nguyen , M. S. Mouksassi , N. Holford , et al., “Model Evaluation of Continuous Data Pharmacometric Models: Metrics and Graphics,” CPT: Pharmacometrics & Systems Pharmacology 6 (2017): 87–109. 27884052 10.1002/psp4.12161 PMC5321813

[25] K. Fearon , F. Strasser , S. D. Anker , et al., “Definition and Classification of Cancer Cachexia: An International Consensus,” Lancet Oncology 12 (2011): 489–495. 21296615 10.1016/S1470-2045(10)70218-7

[26] Project Data Sphere . https://data.projectdatasphere.org/projectdatasphere/html/home. accessed May 21, 2025.

[27] X. S. Xu , M. N. Samtani , A. Dunne , P. Nandy , A. Vermeulen , and F. De Ridder , “Mixed‐Effects Beta Regression for Modeling Continuous Bounded Outcome Scores Using NONMEM When Data Are Not on the Boundaries,” Journal of Pharmacokinetics and Pharmacodynamics 40 (2013): 537–544. 23645382 10.1007/s10928-013-9318-0

[28] M. Reck , F. Taylor , J. R. Penrod , et al., “Impact of Nivolumab Versus Docetaxel on Health‐Related Quality of Life and Symptoms in Patients With Advanced Squamous Non–Small Cell Lung Cancer: Results From the CheckMate 017 Study,” Journal of Thoracic Oncology 13 (2018): 194–204. 29129758 10.1016/j.jtho.2017.10.029

[29] R. Zaim , K. Redekop , and C. A. Uyl‐de Groot , “Analysis of Patient Reported Outcomes Included in the Registrational Clinical Trials of Nivolumab for Advanced Non‐Small Cell Lung Cancer,” Translational Oncology 20 (2022): 101418. 35429903 10.1016/j.tranon.2022.101418 PMC9034386

[30] R. Berman , A. Davies , T. Cooksley , et al., “Supportive Care: An Indispensable Component of Modern Oncology,” Clinical Oncology 32 (2020): 781–788. 32814649 10.1016/j.clon.2020.07.020 PMC7428722

[31] F. Scotté , A. Taylor , and A. Davies , “Supportive Care: The “Keystone” of Modern Oncology Practice,” Cancers 15 (2023): 3860. 37568675 10.3390/cancers15153860 PMC10417474

[32] J. Zhou , A. Cipriani , Y. Liu , G. Fang , Q. Li , and Y. Cao , “Mapping Lesion‐Specific Response and Progression Dynamics and Inter‐Organ Variability in Metastatic Colorectal Cancer,” Nature Communications 14 (2023): 417. 10.1038/s41467-023-36121-y PMC9876906 36697416

[33] J. Zhou , Q. Li , and Y. Cao , “Spatiotemporal Heterogeneity Across Metastases and Organ‐Specific Response Informs Drug Efficacy and Patient Survival in Colorectal Cancer,” Cancer Research 81 (2021): 2522–2533. 33589516 10.1158/0008-5472.CAN-20-3665 PMC8137573

